# Physicians' Views on Utilization of an Electronic Health Record–Embedded Calculator to Assess Risk for Venous Thromboembolism among Medical Inpatients: A Qualitative Study

**DOI:** 10.1055/s-0041-1742227

**Published:** 2022-01-24

**Authors:** Stephanie R. Moss, Kathryn A. Martinez, Cassandra Nathan, Elizabeth R. Pfoh, Michael B. Rothberg

**Affiliations:** 1 Department of Hospital Medicine, Cleveland Clinic Community Care; Department of Pediatric Hospital Medicine, Pediatrics Institute; Cleveland Clinic, Cleveland, Ohio, United States; 2 Center for Value-Based Care Research, Cleveland Clinic Community Care, Cleveland Clinic, Cleveland, Ohio, United States; 3 Brandeis University, Waltham, Massachusetts, United States

**Keywords:** venous thrombosis, deep vein thrombosis, prophylaxis, clinical decision support

## Abstract

**Background** Venous thromboembolism (VTE) causes preventable in-hospital morbidity. Pharmacologic prophylaxis reduces VTE in at-risk patients but also increases bleeding. To increase appropriate prescribing, a risk calculator to guide prophylaxis decisions was developed. Despite efforts to promote its use, providers accessed it infrequently.

**Objective** This study aimed to understand provider perspectives on VTE prophylaxis and facilitators and barriers to using the risk calculator.

**Design** This is a qualitative study exploring provider perspectives on VTE prophylaxis and the VTE risk calculator.

**Participants** We interviewed attending physicians and advanced practice providers who used the calculator, and site champions who promoted calculator use. Providers were categorized by real-world usage over a 3-month period: low (<20% of the time), moderate (20–50%), or high (>50%).

**Approach** During semistructured interviews, we asked about experiences with VTE, calculator use, perspectives on its implementation, and experiences with other risk assessment tools. Once thematic saturation was reached, transcripts were analyzed using content analysis to identify themes.

**Results** Fourteen providers participated. Five were high utilizers, three were moderate utilizers, and six were low utilizers. Three site champions participated. Eight major themes were identified as follows: (1) ease of use, (2) perception of VTE risk, (3) harms of thromboprophylaxis, (4) overestimation of calculator use, (5) confidence in own ability, (6) underestimation of risk by calculator, (7) variability of trust in calculator, and (8) validation to withhold prophylaxis from low-risk patients.

**Conclusions** While providers found the calculator is easy to use, routine use may be hindered by distrust of its recommendations. Inaccurate perception of VTE and bleeding risk may prevent calculator use.

## Introduction

Venous thromboembolism (VTE) causes preventable morbidity among hospitalized patients. 1 2 Multiple national guidelines recommend risk assessment and appropriate thromboprophylaxis for prevention of VTE. 3 4 The National Quality Forum adopted a quality measure requiring all patients to be assessed for VTE risk and recommends that all patients not at low risk for VTE receive thromboprophylaxis, unless contraindicated. 5

There is less guidance about what constitutes low risk, and historically compliance with VTE prophylaxis was poor. 6 Consequently, the Agency for Healthcare Research and Quality (AHRQ) published a guideline advocating for near universal prophylaxis. 7 Similarly, the Joint Commission instituted a core measure requiring either prophylaxis or documentation as to why it was withheld. 8 Yet less attention has been paid to the appropriateness of prophylaxis. Both underuse and overuse of thromboprophylaxis have been documented for medical inpatients. 9 Studies have demonstrated harms of universal prophylaxis, including increased risk of bleeding, 10 11 local discomfort, and heparin-induced thrombocytopenia (HIT), and some have cast doubt on the effectiveness of VTE prophylaxis. 12 13 Therefore, it is important to identify which patients are at risk for VTE and limit prophylaxis to these patients.

**Fig. 1:**
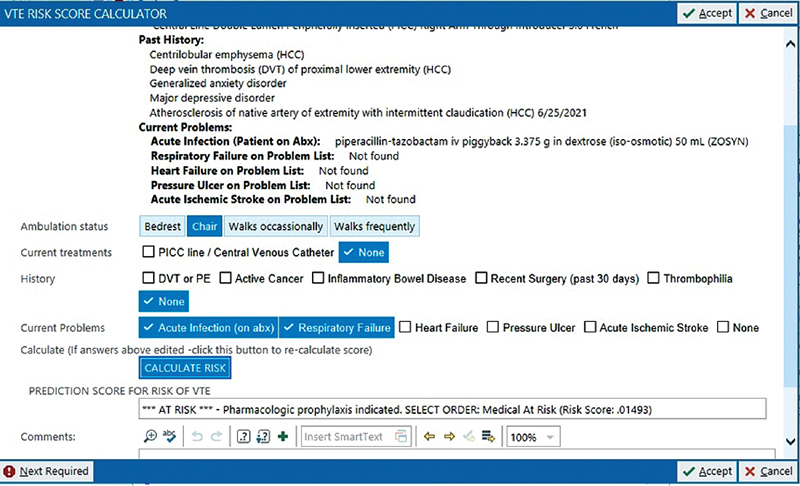
Venous thromboembolism risk calculator within electronic health record.

Many VTE risk assessment tools exist. However, a recent systematic review of eight such tools concluded that none was preferred over the others. 14 To address limitations of prior tools, researchers at our institution created a novel VTE risk calculator based on 5 years of local data from medical inpatients. The purpose of the calculator was to improve appropriate prescription of VTE prophylaxis by identifying patients at low risk for VTE, for whom the harms of prophylaxis outweigh the benefit, while still providing appropriate prophylaxis to at risk patients. In preparation for a pragmatic randomized trial, the calculator, which autopopulates with data from the electronic health record (EHR), was embedded in all admission order sets at the appropriate point in the workflow and was launched by a hyperlink ( Fig. 1 ). There were no pop-up reminders. It was pilot tested in several iterations and used extensively by the piloting physicians who found it helpful. The randomized trial of the calculator was registered with clinicaltrials.gov (registration number: NCT03243708) and began in 2018.

Multiple strategies were employed to promote use of the calculator, including in-person education and monthly reminder e-mails which included details about the derivation and validation of the calculator. At each hospital within our health care system, physician champions promoted the calculator through staff meetings and individual outreach to providers. The study team provided the site champions with weekly audits of their physicians, so they could discuss performance. Despite these efforts, utilization of the calculator remained lower than anticipated. In the 5 months after the calculator was introduced, utilization by site varied from 4 to 68%. Only three of 10 sites achieved >30% utilization.

To understand why the risk calculator was not more widely adopted, we performed a qualitative study to elicit the perspectives of physicians, advanced practice providers (APPs), and site champions and thereby generate hypotheses regarding barriers and facilitators to its use. Although responses were specific to this calculator, they offer insight into VTE prophylaxis more generally and broader adoption of locally derived clinical decision support.

## Methods

### Design

Qualitative inquiry is an important first step in understanding phenomena for which little empirical evidence exists. In this case, we wanted to investigate what factors promoted or hindered adoption of an EHR-based, locally derived risk calculator. To do this, we assumed a constructivist approach and conducted interviews with providers who had the opportunity to use the calculator over a 3-month period, including physicians who served as site champions. We used qualitative content analysis to develop explanatory themes related to uptake of the calculator.

### Participants and Setting

Study participants included attending physicians and APPs working in the Department of Hospital Medicine (DHM) at Cleveland Clinic main campus and regional campuses (nine sites). Despite its international reputation, the Cleveland Clinic's medical services primarily serve a local population of typical general medicine patients. We conducted two rounds of interviews. First, we identified providers who had at least 10 opportunities to use the calculator within a 3-month period to ensure that participants would be sufficiently familiar with the calculator. We categorized providers by level of utilization: low (<20% of the time), moderate (20–50% of the time), or high (>50% of the time). Initially, a random sample of providers with approximately equal numbers in each group were approached. Approximately halfway through the study, we transitioned to a convenience sample of providers who mainly worked at the main campus location, still with representation from all three utilization levels, as these providers were more readily responsive to the e-mail invitation. Based on prior work at our institution, we estimated that approximately 15 interviews would be required to achieve thematic saturation. 15 For the second round, we approached a random sample of site champions to understand site level barriers and facilitators to calculator use.

For both rounds, we invited eligible providers via e-mail to participate in 30-minute semistructured interviews. The first round of interviews were conducted from April to July 2019, either in person or by phone. The second round was conducted by phone between March and April 2020. Cleveland Clinic's Institutional Review Board (IRB) approved this study (identifier no.: 19–457).

### Interview Guide Development

The interview guide ( Supplementary Appendix A ) was developed with input from study team members. Prompts were designed to focus on perceived benefits and barriers to calculator use. We grouped prompts in the interview guide by the following sections: beliefs and experiences with VTE in the hospital, calculator-specific questions, and use of risk prediction tools generally. We developed a separate interview guide for site champions ( Supplementary Appendix B ) to better understand the challenges they faced in promoting the use of the calculator with the following sections: successes and barriers in the role of site champion, personal use of the VTE calculator, perceptions of colleagues' calculator utilization, and concerns.

### Data Collection

The first author (S.R.M.) conducted all interviews. She is an attending physician in the DHM and previously known to most study participants as a colleague. As a researcher, she made deliberate effort to avoid making assumptions based on her own beliefs on the subject. After obtaining verbal consent, a digital recorder was used to audio record the interviews which were anonymized and recorded by number. Interviews were then transcribed verbatim. Patients were not compensated for their participation.

### Data analysis

Two team members (S.R.M. and C.N.) independently analyzed the interviews using open and axial coding to identify themes. Analysis was performed manually. The entire team systematically discussed categorization of themes to identify broader patterns as an iterative process. As we were interested in understanding if certain themes explained differences in calculator utilization, we assessed differences in participant responses by real-world calculator use. We assessed that data saturation had been achieved when no new codes or themes were generated from the interviews as has been previously described. 16 Once thematic saturation was achieved, no further interviews were performed. During this process for the first round of interviews, the team recognized that site champions could provide additional insight based on initial themes. This led to the second round of interviews with site champions specifically. Once the team had completed the identification and sorting of themes, two authors (S.R.M. and M.B.R.) rereviewed the transcripts for “goodness-of-fit.”

## Results

For the first round of interviews, there were 1,007 eligible providers. Fifty-six providers targeting representation from all three levels of calculator utilization were contacted for participation. Fourteen providers, including 12 physicians and 2 APPs, 9 males and 5 females, were participated. Five were high utilizers of the calculator, three were moderate utilizers, and six were low utilizers. Thirteen worked at least partly at the main campus. For the second round, 9 of 20 site champions were randomly contacted, and we interviewed three site champions, all of whom worked at regional locations, one of which was a higher utilizing center and two were lower utilizing.

We identified eight themes in the first round of provider interviews, and two additional themes identified from site champion interviews ( Table 1 ).

**Table 1:** Results: themes encompassing facilitators and barriers of VTE calculator use

Theme	Quotes	
Ease of use	“It's easy to use when you admit someone. (3-mod) a “I think it probably takes five seconds … easy to use, quick.” (8-mod) “I think it was very easy to use, and it was kind of straightforward.” (9-low) “It doesn't give you too many prompts, but … everyone is just getting more and more prompts and it just slows you down.” (12-low) “It should be there to help you out if needed as part of the order set, but it shouldn't be mandatory.” (7-high)	
Perception of HA-VTE risk	Perceived HA-VTE risk lower: “I know they happen, but I'm not sure the percentage because I feel like I don't really see that many myself.” (1-high) “Maybe a handful of times out of 12 years.” (3-mod) “I don't think it's that common as we make them out to be.” (10-low) “The risk is like less than a half [percent].” (6-high) “Less than 1% I see.” (11-low)	Perceived HA-VTE risk higher: “Very common … I've had multiple patients over the past few years who forgone VTE prophylaxis and invariably ended up with a clot.” (2-mod) “Between 5 to 10%.” (5-low)
Harms of thromboprophylaxis	Underestimation of harms: “No, there's no disadvantages (of pharmacologic prophylaxis).” (2-mod) “I don't think I've ever encountered any significant…bleeding with just prophylaxis, so no.” (9-low)	Appreciation of harms: “Pain coming from the injection, and then prolonged hospital stay due to complications…like they develop HIT.” (1-high) “Certainly patient comfort…you get a lot of bruising.” (3-mod) “Definitely increased risk of bleeding, HIT, and delay of procedures.” (4-high) “… (he) was exposed to heparin, came back, was exposed to heparin again, we didn't realize it … he developed full blown HIT … and literally clots everywhere. It was just awful.” (10-low)
Estimation of own calculator usage	Overestimation of utilization: “I use it for every admission that I do.” (1-high) “I would recommend using it all the time and I try to use it all the time.” (3-mod) “Over the last 6 months I've been doing it more than 60% … and I aim for 100% because I like it.” (12-low)	Acknowledgment of low utilization: “I've actually probably used it maybe once or twice.” (9-low) “I would say like 20%. Maybe even less.” (10-low)
Confidence in own ability to assess VTE risk	High confidence: “I'm very confident in doing a clinical assessment, and you know, a lot of those factors are included in the calculator, but clinically I am very confident in assessing risk.” (2-mod) “I feel very confident … I think just assessing what the risks factors are and knowing some of the risk factors that go along with increased risks of VTE. So I feel like just being aware of them, I feel confident in thinking that they would need prophylaxis.” (7-high)	Qualified confidence: “I feel fairly confident … (the calculator) helps me feel like I'm making the right decision.” (1-high) “I think that I probably overestimate the risk of VTE.” (4-high) “… maybe I'm not a good judge of determining the risk …” (6-high)
Underestimation of VTE risk by calculator	“There have been actually a lots of times for the VTE risk calculator … there's no prophylaxis indicated and that this patient is low risk, but just based on … clinical judgment I feel like this is someone who probably still needs to get prophylaxis.” (7-high) “certain other patients … it's a gut instinct, I feel are at a higher risk and I'm just not comfortable with what the tool says … so sometimes … I just put them on a DVT prophylaxis.” (10-low)	
Variability of trust in calculator's development	Doubt: “I haven't seen the evidence of the Cleveland Clinic VTE risk calculator. What's the evidence, why did they generate it …” (2-mod) “Have you validated this? I don't think so, correct?” (10-low)	Trust: “I pretty much trusted my colleagues. They said it's good then, you know, we'll do it.” (3-mod) “… we've got frequent updates about it, we've had talks about it, we've had presentations …” (6-high)
Calculator can support withholding pharmacologic prophylaxis in low-risk patients	Give less prophylaxis: “… with the risk calculator I've been able to hopefully appropriately select fewer patients who need pharmacologic therapy.” (4-high) “I think I have used less heparin (subcutaneous).” (1-high) “I think I order (prophylaxis) less than I would have in the past.” (6-high)	No change/near-universal prophylaxis: “I order VTE prophylaxis for almost everybody who is above the age of 40, invariably.” (2-mod) “I would think pharmacological prophylaxis is probably 90 + %.” (5-low) “For pharmacologic, I would say maybe it will be high actually, probably 80%.” (9-low)

Abbreviations: HA-VTE, hospital-associated venous thromboembolism; HIT, heparin induced thrombocytopenia; VTE, venous thromboembolism.

a Quotes are identified by the interviewee (1 thru 14) and the level of utilization of the calculator by the interviewee (low, moderate, and high).

### Theme 1. Ease of Use

Most participants reported that the calculator was easy to use and did not add much time or interfere with their workflow. However, it was acknowledged that even small tasks can impact work flow and could lead to some providers ignoring the calculator. Most participants did not favor a “hard stop” requiring completion of the calculator.

### Theme 2. Perception of Hospital-Associated Venous Thromboembolism Risk

Providers varied in their estimates of hospital-associated VTE incidence. Most providers accurately assessed the risk as low; others felt the risk was higher. Some of the providers who overestimated the numerical incidence still felt the risk was low qualitatively.

### Theme 3. Harms of Thromboprophylaxis

Some providers underestimated the harms of pharmacologic prophylaxis. Those who did appreciate harms cited discomfort and inefficiency of clinical care in addition to bleeding and thrombocytopenia.

### Theme 4. Overestimation of Own Calculator Usage

Regardless of actual usage, most providers overestimated their calculator use. High and moderate users believed that they frequently, if not always, used the calculator, and some low utilizers also overestimated their usage. However, low utilizers were more likely to admit that they did not use the calculator.

### Theme 5. Confidence in Own Ability to Assess Venous Thromboembolism Risk

Most providers were reasonably confident in their ability to assess VTE risk. Those who expressed significant distrust in the VTE risk calculator were particularly confident in their own abilities. However, other providers who trusted the calculator expressed doubt.

### Theme 6. Underestimation of Venous Thromboembolism Risk by Calculator

Many providers felt that the calculator underestimated some patients' risk of VTE. Most providers recounted instances where the calculator indicated that the patient was at low risk but they disagreed.

Specifically, some providers felt that certain risk factors were missing from the calculator (e.g., oral contraceptive use and inflammatory bowel disease), including some risk factors which were actually in the calculator (e.g., tobacco use), indicating that some providers were not familiar with calculator inputs.

### Theme 7. Variability of Trust in the Calculator's Development

Despite repeated attempts by the team who developed the calculator to educate providers about its development and validation, some providers did not trust it. Moderate and high utilizers generally expressed trust in both the calculator and their colleagues.

All three site champions interviewed stated inaccurately that the calculator was derived from data at hospitals nationally when it was actually derived from data within their own health care system.

### Theme 8. Calculator Can Support Withholding Pharmacologic Prophylaxis in Low-Risk Patients

High and moderate utilizers generally expressed that the calculator validated their decision to withhold VTE prophylaxis from low-risk patients. Those who felt that the calculator had changed their practice reported prescribing less pharmacologic prophylaxis. Conversely, low utilizers and those who distrusted the calculator often reported giving prophylaxis almost universally.

### Site Champion Perspectives on Reasons for Variation in Venous Thromboembolism Calculator Use

Site champions were aware of their site's adherence. “It became a bit of a competition … not among just the internal providers but … all the other sites, so every time we got the curve …, we beat (other hospital name).” [site champion 1] “A few of us would do it on a regular basis but that was not enough, so I constantly reached out … weekly, monthly, to everybody, but it was quite challenging.” [site champion 3]

Larger facilities and presence of private physicians were identified as barriers to routine calculator use. “I think in the smaller facility it's probably … easier to do …(because) we work with these folks every day …, so we kind of grow up together.” [site champion 1] “As far as the private (physician) group is concerned …, they were not receptive …, they were polite enough to listen to me, but … they made me feel that I was wasting their time.” [site champion 2] “Fairview is big and you know it's kind of 50/50 where 50% … they're all the private doctors, so it's quite big, so that's challenging, but Avon is small, so I think reaching out to people is easier.” [site champion 3]

Local culture regarding quality initiatives more broadly appeared to play a role in calculator adoption. “I also work nights at (hospital name), and I saw them, those providers doing that on a regular basis.” [site champion 3] “Avon compared to Fairview, I note that they really follow the rules, like even problem based notes, everybody follows that, everybody follows what DHM wants at Avon, and I think, … (the local director), she's pretty strong, you have to follow it.” [site champion 3]

## Discussion

Despite their promise to transform care, EHR-based decision-support tools are rarely used in clinical practice. In this qualitative, hypothesis-generating study of provider perspectives of barriers and facilitators to implementation and use of an EHR-embedded locally derived VTE risk calculator, we identified eight themes which offered insight into the way physicians approach VTE and the risk calculator. Providers were divided over how much to trust the calculator, confidence in their own clinical judgment, the risk of VTE in medical patients, and the harms of prophylaxis. Trust in the calculator's predictions, accurate perception of the risk of VTE as low, and appreciation of the harms of thromboprophylaxis-promoted calculator use. Lack of trust in the calculator, confidence in personal clinical judgment, the belief that almost all patients need prophylaxis, and under appreciation of harms tended to discourage calculator use.

Studies suggest that hospital-associated VTE occurs in approximately 1% of medical inpatients, with most cases detected after discharge. 11 12 17 18 19 During the randomized trial of our VTE risk calculator, the 14-day risk of VTE was 0.9%, indicating that our patient population has a similar VTE risk to that of medical inpatients nationally. We found that individual providers varied considerably in their assessment of hospital-associated VTE occurrence, some noting they had seen only a handful of cases, while others believed it occurred in as many as 10% of admitted patients. Overestimating the risk of VTE and believing all patients require prophylaxis would make the calculator less valuable. Similarly, if there were no harms associated with prophylaxis, there would be little reason not to give it and no need for a calculator. Thus, overestimation of the benefit of prophylaxis and underestimation of harms would obviate the need to assess risk. While these are the very biases that the calculator was designed to correct, those who would most benefit from the calculator would find least reason to use it.

Most high and moderate utilizers expressed trust in the calculator. They recalled communication providing information on the calculator's derivation and validity or expressed implicit trust in their colleagues. Others expressed distrust, perceiving that the calculator was inadequately validated. Concern that important risk factors were omitted may have fed this skepticism. Distrustful providers also reported confidence in their own ability to assess VTE risk. Most of these were low utilizers, although one was moderate and one was high. These last two expressed that they felt forced to use the calculator because of the audit and feedback, but they did not follow the calculator's recommendation. Skepticism regarding the calculator's validity paired with high confidence in personal clinical judgment offered complementary reasons to forgo it. Publishing the data on the risk calculator prior to the randomized trial might have improved faith in it, but only if providers were aware of the publication. Those developing localized risk models for other conditions should consider the challenge of legitimacy, especially if local physicians already feel confident in their abilities to predict risk.

When providers' clinical assessment disagreed with the calculator, providers generally erred on the side of giving prophylaxis, especially if they perceived the risks of prophylaxis to be low. Pressure from peers, fear of malpractice, and hospital quality measures may motivate physicians to order prophylaxis broadly, including for patients who do not require it. 20 21 22 One reported benefit of the calculator was offering support for physicians' decisions to withhold prophylaxis when they felt it was not indicated, leading to less prescribing.

Past studies of clinical decision tools embedded in the EHR have shown similar variability in uptake. For example, the Patient Risk Information Services Manager (ePRISM) tool for estimating bleeding, restenosis, and mortality risk among patients undergoing percutaneous coronary intervention improves patient satisfaction with the informed consent process and reduces bleeding events. 23 However, in a large prospective cohort study, many physicians did not use it to guide their decision-making and there was significant hospital level variability. 23 A qualitative evaluation of physicians' perspectives on the tool found that physicians did not use ePRISM because they preferred to rely on their personal experience, felt their own decision-making was sound, and worried that the model would lead to patients not being offered a potentially beneficial therapy. 24 Similarly, we found providers often preferred to rely on their clinical judgment, particularly when they distrusted the calculator.

Although extra clicks are seldom welcome in clinical care, providers in our study reported the calculator was easy to use and did not substantially interrupt their workflow. Nevertheless, most did not use it often, because they usually did not find the information valuable. The finding that many providers overestimated the extent to which they used the calculator suggests that official usage data may underestimate actual usage. To register the usage in the EHR, providers had to hit “submit” after entering their data. Some may have used the calculator without it registering.

Interviews with site champions provided additional insight into facilitators and barriers to usage. Site champions found it easier to promote usage at hospitals with fewer providers. In particular, site champions struggled with engaging physicians who were not hospital employees and may therefore be less receptive to hospital research or quality initiatives. Hospital culture and a group's attitude toward quality initiatives was an important determinant of compliance.

Communicating change throughout a large health system with hundreds of physicians spread across 10 hospitals is difficult. Despite multiple attempts, both in person and via e-mail, to explain the development of the calculator, distrust was common. Even site champions were unsure of the calculator's derivation which likely affected their messaging. This highlights the difficulty of communicating with providers who are barraged with daily e-mails, pages, phone calls, and text messages. One possible solution might be point-of-care education in which the rationale and validation information can be accessed from the calculator itself.

## Limitations

Our study has several limitations. As we were not able to achieve a truly random sample, selection bias may have occurred. Although we reached thematic saturation based on analysis of 17 total participants (including site champions), it is possible that our results would have been different if we had spoken to a different population. Providers with more positive views of the calculator may have been more likely to respond to interview requests, compared with providers who were either ambivalent or felt negatively. This may have limited our understanding of barriers to uptake. Finally, the majority of respondents work at the main campus and respondents did not represent all clinical sites; no private physicians responded to interview requests. However, site champions interviewed represented three different regional sites.

## Conclusion

The widespread adoption of EHRs and the ability of health systems to develop local risk calculators creates the potential to deliver personalized care to millions of hospitalized patients. To date, however, despite the development of thousands of risk prediction models, few have been implemented in routine clinical care. Our study provides general insight into the difficulty of introducing such models, as well as specific understanding of providers' perceptions of VTE risk assessment. Improving physicians' understanding of the prevalence of VTE, as well as the harms of prophylaxis, while addressing concerns regarding the validity of the calculator may result in greater uptake. Our study highlights the difficulties in disseminating an intervention throughout numerous hospitals which may require customized strategies to support adoption, taking into account each hospital's culture and workforce.
