# Supplementary material for: Physicians' Views on Utilization of an Electronic Health Record–Embedded Calculator to Assess Risk for Venous Thromboembolism among Medical Inpatients: A Qualitative Study
Source: TH Open. 2022 Jan 24;6(1):e33–9. doi: 10.1055/s-0041-1742227 (PMC8786561; doi:10.1055/s-0041-1742227)
Supplement: Supplementary file 1 — Supplementary Material [file 10-1055-s-0041-1742227-s210058.pdf]

Supplementary Appendix A

Introduction

Hello Dr. ___, my name is ___________ and I am one of the hospitalists. I am conducting a research study with the Cleveland Clinic Center for Value-Based Research. We want to understand how physicians think about venous thromboembolism (VTE) risk assessment for their patients, and get their perspective on the VTE risk calculator, which was introduced into the electronic health record in 2018.

I would like your permission to audio record the interview so that I can accurately document the information you share with me today. All of your responses are confidential. As a reminder, your participation is completely voluntary and you can stop participating at any time. Thank you for your participation in this study.

I would like to start by hearing about your experiences with VTE.

Prompts:
How common a complication is hospital acquired VTE for the patients you see? (<5, 10, or 20%)
What are some patient characteristics or clinical situations that prompt you to worry about VTE? Please explain.

When do you consider ordering VTE chemoprophylaxis?

Prompts if needed:
What patient characteristics?
What disease characteristics?

What do you consider to be the benefits of prophylaxis?
What factors do you feel are contraindications to VTE prophylaxis?

Prompts if needed:
What patient characteristics?
What disease characteristics?
What do you consider the disadvantages or harms of thromboprophylaxis?
How many of your patients (%) do you order VTE prophylaxis for? (<10, 25, 50, or >75%)
How confident are you in your ability to assess a patient's risk for VTE?

Now I want to transition to asking you about the VTE calculator.

Have you seen or heard about the VTE risk calculator? *(If not, explain what the VTE calculator is and where to find it.)*
Have you used the VTE risk calculator?

If yes:
For approximately what percentage of your patients do use the calculator? (<10, 25, 50, or 75%)
Please describe when you typically use the calculator
How has the tool changed your prescribing practice for VTE prophylaxis?
What conversations (if any) do you have with patients regarding VTE prophylaxis? Has the calculator affected this?
How has the calculator impacted your clinical work flow?
Have you encountered a situation where your clinical judgment and the VTE risk calculator didn't align?
If yes: What did you do in this situation and why?
If no:

What are the reasons you do not use the tool?

Prompts if needed:
Do you find the tool difficult to find or use (too long or difficult to find)?
Do you have concerns about the validity of the tool to identify patients at risk for VTE?
What is your experience with other risk assessment tools for VTE prophylaxis (Padua's score and Caprini's score)?
How confident are you in the calculator to accurately assess patient risk for VTE?
What do you feel are the benefits and/or harms of the calculator?
Are there certain situations when you use the calculator and others when you don't? Please describe.

Prompts if needed:
Is it hard to remember to use?
Are there certain risk factors you feel are missing from the calculator?
What challenges have you encountered with using the calculator?
What percentage of your colleagues do you think are using the calculator (<10, 25, 50, or 75%)?
What barriers to use of the calculator do you think your colleagues would site?
Were you aware that each campus has a local champion for the calculator?

If yes:
How did having a local champion influence your utilization of the calculator?
How would you feel if there were a hard stop in the EHR requiring you to use the calculator prior to signing admission orders?

Now I'd like to get your thoughts on risk prediction tools more generally.

Do you regularly use any other risk prediction tools in your practice?

If yes:
Which ones? (CHADS2VASC, Wells, or HASBLED)
How do you decide which ones to use?
If no:
What concerns about risk prediction tools do you have that prevent you from using them?

 © 2022. The Author(s).

Finally, we would like your input on how to improve the VTE calculator.

What do you like about the VTE calculator?

What would you change about the VTE calculator?

Ease of use?

Location of the calculator?

Risk factors included?

Finally, I wanted to leave you the opportunity to share any additional thoughts about the VTE risk calculator.

Thank you very much for your time and thoughts.

Supplementary Appendix B

Interview Questions for Site Champions

Introduction

Hello Dr. __________, my name is ________________ and I am one of the hospitalists. I am conducting a research study with the Cleveland Clinic Center for Value-Based Research. We are studying use of the venous thromboembolism (VTE) risk assessment calculator. I would like to get your perspective the calculator and its utilization given your role as a site champion for the calculator when it was rolled out.

I would like your permission to audio record the interview so that I can accurately document the information you share with me today. All of your responses are confidential. As a reminder, your participation is completely voluntary and you can stop participating at any time. Thank you for your participation in this study.

I'd like to start by asking you some questions about your role as site champion.

Tell me about your role as a site champion

Prompts if needed:

Did you send emails? Did you speak to colleagues collectively and/or individually?

At which sites did you promote the VTE calculator?

What method of engaging with your colleagues was the most successful? Least?

On as scale from 1-10, how effective where you as a site champion? Why did you rate yourself that number?

Now I'd like to ask you some questions about your own use of the VTE calculator.

How confident are you in the calculator to accurately assess patient risk for VTE?

What are the benefits of the calculator? Harms?

To what extent has the VTE risk calculator changed your own prescribing practice of VTE prophylaxis?

Have you encountered a situation where your clinical judgment and the VTE risk calculator didn't align?

If yes: what did you do in this situation and why?

How has the calculator impacted your clinical work flow?

Lastly, I'd like to ask you some questions about your colleagues' use of the calculator.

What did your colleagues say when you talked to them about the VTE calculator?

What barriers to use of the calculator were mentioned? Advantages?

What concerns do you think your colleagues had about using the calculator?

If low use site:

What factors do you think prevented more of your colleagues from using the calculator?

If high use site:

What factors do you think facilitated your colleagues adopting the calculator into their practice?

Some clinicians who were interviewed expressed concern about the validation of the calculator. How do you feel about the validation data behind the VTE risk calculator?

While you were a site champion, did your colleagues ask you about the validation of the calculator?

Do you feel there were any logistical issues with the calculator that hindered its adoption?

Prompts if needed:

Do you find the tool difficult to find or use?

What would you change about the VTE calculator?

Ease of use? Location of calculator? Risk factors included?

Finally, I wanted to leave you the opportunity to share any additional thoughts about the VTE risk calculator.

Thank you very much for your time and thoughts.

 © 2022. The Author(s).
